# Associations with Intraocular Pressure in a Large Cohort: Results from the UK Biobank

**DOI:** 10.1016/j.ophtha.2015.11.031

**Published:** 2016-04

**Authors:** Michelle P.Y. Chan, Carlota M. Grossi, Anthony P. Khawaja, Jennifer L.Y. Yip, Kay-Tee Khaw, Praveen J. Patel, Peng T. Khaw, James E. Morgan, Stephen A. Vernon, Paul J. Foster, UK Biobank Eye and Vision Consortium

**Affiliations:** 1 NIHR Biomedical Research Centre, Moorfields Eye Hospital NHS Foundation Trust and UCL Institute of Ophthalmology, London, United Kingdom; 2 Department of Public Health & Primary Care, University of Cambridge, Cambridge, United Kingdom; 3 School of Optometry & Vision Sciences, Cardiff University, Cardiff, United Kingdom; 4 Department of Ophthalmology, Nottingham University Hospital NHS Trust, Nottingham, United Kingdom

**Keywords:** BMI, body mass index, CCT, central corneal thickness, CI, confidence interval, DBP, diastolic blood pressure, IOP, intraocular pressure, IOPcc, corneal-compensated intraocular pressure, IOPg, Goldmann-correlated intraocular pressure, OAG, open-angle glaucoma, ORA, Ocular Response Analyzer, SBP, systolic blood pressure

## Abstract

**Purpose:**

To describe the associations of physical and demographic factors with Goldmann-correlated intraocular pressure (IOPg) and corneal-compensated intraocular pressure (IOPcc) in a British cohort.

**Design:**

Cross-sectional study within the UK Biobank, a large-scale multisite cohort study in the United Kingdom.

**Participants:**

We included 110 573 participants from the UK Biobank with intraocular pressure (IOP) measurements available. Their mean age was 57 years (range, 40–69 years); 54% were women, and 90% were white.

**Methods:**

Participants had 1 IOP measurement made on each eye using the Ocular Response Analyzer noncontact tonometer. Linear regression models were used to assess the associations of IOP with physical and demographic factors.

**Main Outcome Measures:**

The IOPg and IOPcc.

**Results:**

The mean IOPg was 15.72 mmHg (95% confidence interval [CI], 15.70–15.74 mmHg), and the mean IOPcc was 15.95 mmHg (15.92–15.97 mmHg). After adjusting for covariates, IOPg and IOPcc were both significantly associated with older age, male sex, higher systolic blood pressure (SBP), faster heart rate, greater myopia, self-reported glaucoma, and colder season (all *P <* 0.001). The strongest determinants of both IOPg and IOPcc were SBP (partial *R*^2^: IOPg 2.30%, IOPcc 2.26%), followed by refractive error (IOPg 0.60%, IOPcc 1.04%). The following variables had different directions of association with IOPg and IOPcc: height (−0.77 mmHg/m IOPg; 1.03 mmHg/m IOPcc), smoking (0.19 mmHg IOPg, −0.35 mmHg IOPcc), self-reported diabetes (0.41 mmHg IOPg, −0.05 mmHg IOPcc), and black ethnicity (−0.80 mmHg IOPg, 0.77 mmHg IOPcc). This suggests that height, smoking, diabetes, and ethnicity are related to corneal biomechanical properties. The increase in both IOPg and IOPcc with age was greatest among those of mixed ethnicities, followed by blacks and whites. The same set of covariates explained 7.4% of the variability of IOPcc but only 5.3% of the variability of IOPg.

**Conclusions:**

This analysis of associations with IOP in a large cohort demonstrated that some variables clearly have different associations with IOPg and IOPcc, and that these 2 measurements may reflect different biological characteristics.

Elevated intraocular pressure (IOP) is one of the most significant risk factors for the development^1^ and progression^2^ of open-angle glaucoma. Intraocular pressure is a multifactorial trait with a heritability of 29% to 62%.^3^, ^4^ Many epidemiologic studies have examined the association of IOP with physical and sociodemographic factors across different populations, and these factors have been shown to account for approximately 10% of IOP variability.^5^, ^6^, ^7^, ^8^ Although some associations with IOP have been demonstrated consistently, such as systolic blood pressure (SBP),^7^, ^8^, ^9^, ^10^ other factors such as age^7^, ^8^, ^11^, ^12^ and sex^7^, ^8^, ^9^, ^11^, ^13^ have a less consistent effect. There is also growing evidence that corneal biomechanics influence IOP measurements.^14^, ^15^, ^16^ The UK Biobank is one of the largest prospective cohort studies with ocular data globally and will lend statistical power to detecting weaker associations of IOP. In this study, we explore the associations of both Goldmann-correlated IOP (IOPg) and corneal-compensated IOP (IOPcc) measured by the Ocular Response Analyzer noncontact tonometer (ORA).

## Methods

The UK Biobank is a large-scale multisite cohort study established by the Wellcome Trust medical charity, Medical Research Council, Department of Health, Scottish Government, and Northwest Regional Development Agency. The overall study protocol (http://www.ukbiobank.ac.uk/resources/) and protocols for individual tests (http://biobank.ctsu.ox.ac.uk/crystal/docs.cgi) are available online. In brief, an extensive baseline questionnaire, physical measurements, and biological samples were undertaken in 22 assessment centers between 2006 and 2010. All UK residents aged 40 to 69 years who were registered with the National Health Service and living up to 25 miles from 1 of the 22 study assessment centers were invited to participate. The work was carried out with the approval of the North West Research Ethics Committee (reference number 06/MRE08/65), in accordance with the principles of the Declaration of Helsinki.

Ophthalmic data were collected in late 2009 in 6 assessment centers as an additional enhancement to the initial baseline assessment. These 6 centers are distributed widely across the United Kingdom, including Croydon and Hounslow in Greater London, Liverpool and Sheffield in Northern England, Birmingham in the Midlands, and Swansea in Wales. Participants completed a touch-screen self-administered questionnaire on their general health and socioeconomic status. The Townsend deprivation index was determined according to the participants' postcodes at recruitment and the corresponding output areas from the preceding national census. The index was calculated on the basis of the output area's employment status, home and car ownership, and household condition; the higher and more positive the index, the more deprived an area. The choices for ethnicity include white (English/Irish or other white background), Asian or British Asian (Indian/Pakistani/Bangladeshi or other Asian background), black or black British (Caribbean, African, or other black background), Chinese, mixed (white and black Caribbean or African, white and Asian, or other mixed background), or other ethnic group (not defined). Smoking status was determined by the participant's answer to “Do you smoke tobacco now?,” from the selection of yes, on most or all days/only occasionally/no/prefer not to answer. Diabetes status was determined as those who answered yes to “Has a doctor ever told you that you have diabetes?” Glaucoma and macular degeneration statuses were determined as those who selected “glaucoma” or “macular degeneration” from a list of eye disorders to the question, “Has a doctor told you that you have any of the following problems with your eyes?”

### Measurements

Blood pressure and heart rate were measured using the HEM-70151T digital blood pressure monitor (Omron, Hoofddorp, The Netherlands). Two measurements of each were taken, and the mean was used in subsequent analysis. Weight was measured with the BV-418 MA body composition analyzer (Tanita, Arlington Heights, IL). Height was measured using a Seca 202 height measure (Seca, Birmingham, UK). Body mass index (BMI) was calculated as weight (kg)/height (m)^2^. Waist circumference at the level of the umbilicus was measured using a Wessex nonstretchable sprung tape measure. Autorefraction was performed using an RC5000 Auto Refkeratometer (Tomey, Nagoya, Japan), and refractive error (spherical equivalent) was calculated as sphere power + (cylinder power/2). The IOP was measured once for each eye (right eye first) using the ORA (Reichert Corp., Philadelphia, PA), and only 1 measurement per eye was taken. Participants who had eye surgery within the previous 4 weeks or those with possible eye infections were precluded from having IOP measured. The ORA flattens the cornea with a jet of air and uses an electro-optical system to measure the air pressures at which the cornea flattens both inward and outward. The average of the 2 ORA pressure values was calibrated against Goldmann applanation tonometer measures to derive IOPg. The IOPcc was derived using proprietary formulae to correct for the corneal biomechanical properties.^17^

### Statistical Analysis

Left eye IOP values were chosen for the main analyses because they were measured after the right eye and were possibly less prone to artifacts with the participant more familiar with the test. Participants who reported having had laser refractive surgery or corneal graft surgery in the left eye were excluded from the analysis because corneal surgery would bias the relationship between IOPg and IOPcc. Body mass index was examined between 20 and 40 kg/m^2^ (95% of the study population), because BMI outside this range showed a nonlinear relationship with IOP. Smoking status was dichotomized to regular (smokes on most or all days) and current nonsmokers (ex-smokers and never smokers) to maximize the potential to detect an effect. Season of IOP measurement was categorized into spring (March to May), summer (June to August), autumn (September to November), and winter (December to February).

The variables to be examined for associations with IOP were decided a priori on the basis of previous published studies. The possibility of clustering of IOP within each center of assessment was explored, but the intraclass correlation coefficients were very low (0.004 for IOPcc, 0.0005 for IOPg), which indicated that clustering accounted for a very small proportion of the variance in IOP. Therefore, we elected to proceed with multiple regression analysis using the center of assessment as a covariable to account for the potential underlying small differences in associations with IOP. Variations in characteristics between the centers were explored using multiple 1-way analysis of variance with Bonferroni correction for continuous variables and chi-square test for categoric variables.

Associations between IOP and continuous variables were first explored graphically. The relationship with sex, age, Townsend deprivation index, center of assessment, weight, height, waist circumference, SBP and diastolic blood pressure (DBP), BMI, refractive error, smoking status, diabetes, glaucoma, macular degeneration, and season of IOP measurement were explored with univariable linear regression. All examined variables were included in a multivariable regression model. All statistical analyses were performed using STATA (Stata/IC 12.0; StataCorp LP, College Station, TX). A more robust statistical significance threshold of *P <* 0.001 was used to avoid false-positives due to the large number of tests carried out. Further details of the derivation of the variables and missing data can be found on the UK Biobank online data showcase (http://biobank.ctsu.ox.ac.uk/crystal/label.cgi).

## Results

**Figure 1:**
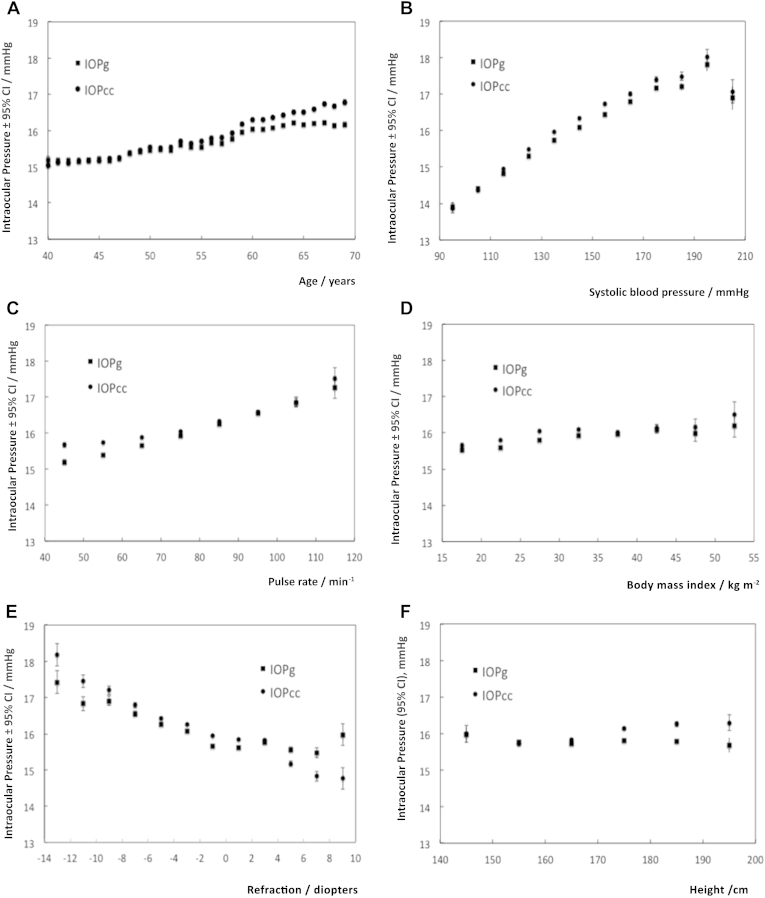
Graphs showing that Goldmann-correlated intraocular pressure (IOPg) and corneal-compensated intraocular pressure (IOPcc) increase linearly with **(A)** age, **(B)** systolic blood pressure, **(C)** pulse rate, **(D)** body mass index (BMI). **(E)** The IOPg and IOPcc show an inverse relationship with refractive error. **(F)** Height has an insignficant relationship with IOPg and IOPcc in univariable regression, but a differential relationship with IOPg and IOPcc in multivariable regression. The error bars represent the 95% confidence intervals (CIs).

Of the 502 656 participants in the whole UK Biobank cohort, 112 690 underwent IOP measurements, and 112 285 had valid measurements. Table 1 summarizes their mean IOP stratified by age, sex, and laterality. Mean IOP was slightly higher in the right eye than the left eye for both IOPg and IOPcc (mean difference, 0.14 mmHg IOPg; 95% confidence interval [CI], 0.12–0.16 mmHg, paired *t* test *P <* 0.001; 0.07 mmHg IOPcc; 95% CI, 0.05–0.09 mmHg; *P <* 0.001). Therefore, left eye values were used in all subsequent analyses because they were measured after the right eye and were possibly less prone to artifacts with the participant more familiar with the test. The mean left IOPg was 15.72 mmHg (95% CI, 15.70–15.74 mmHg), and the mean left IOPcc was 15.95 mmHg (95% CI, 15.92–15.97 mmHg). The IOPg and IOPcc increased linearly with age, SBP, DBP, pulse rate, and BMI (Fig 1A–D) and decreased linearly with refractive error (Fig 1E).

Table 2 summarizes the characteristics of the 110 573 study participants, which excluded those whose left eye has had laser refractive surgery or corneal graft surgery. The completeness of each variable is included in Table 2, which is generally high (98.6%–100% complete). Their mean age was 57.3 years (range, 40–70 years), 54.1% were women, and the majority were white (89.6%). Significant differences between men and women were found for age, distribution of participants among the centers of assessment, ethnicity, deprivation index, height, weight, BMI, waist circumference, SBP, DBP, pulse rate, smoking status, and the percentage with self-reported glaucoma and diabetes.

Among the 6 centers of assessment, mean IOPcc was significantly different (*P <* 0.001, analysis of variance) but not mean IOPg (*P* = 0.046). Specifically, IOPcc was significantly lower in Birmingham than every center except Swansea by 0.05 to 0.41 mmHg (*P <* 0.001). The centers were also different in ethnicity, deprivation index, and season of test (*P <* 0.0001). As a result, the center of assessment was included as a variable in the regression models. Croydon was selected as the baseline center because it contributed the largest number of participants.

The associations of IOP with physio-demographic factors were tested using univariable linear regression stratified by sex (Table 3, Table 4) and multivariable regression (Table 5). All covariates in the univariable model were included in the multiple regression model to allow direct comparisons between IOPg and IOPcc. The DBP and waist circumference were excluded because of collinearity between DBP and SBP, and waist circumference with BMI. After adjusting for covariates, the following were significantly associated with both IOPg and IOPcc: older age (0.18 mmHg IOPg/decade, *P <* 0.001; 0.49 mmHg IOPcc/decade, *P <* 0.001), male sex (0.18 mmHg IOPg, *P <* 0.001; 0.35 mmHg IOPcc *P <* 0.001), SBP (0.035 mmHg IOPg, *P <* 0.001; 0.033 mmHg IOPcc, *P <* 0.001), pulse rate (0.023 mmHg IOPg, *P <* 0.001; 0.018 mmHg IOPcc, *P <* 0.001), myopic refractive error (−0.11 mmHg IOPg/diopter, *P <* 0.001; −0.14 mmHg IOPcc/diopter, *P <* 0.001), self-reported glaucoma (1.97 mmHg IOPg, *P <* 0.001; 2.30 mmHg IOPcc, *P <* 0.001), and colder season (baseline winter; IOPg −0.14 mmHg spring, −0.27 mmHg summer; IOPcc −0.29 mmHg spring, −0.37 mmHg summer, *P <* 0.001). Systolic blood pressure was the most important determinant of both IOPg and IOPcc, accounting for 2.30% and 2.26% (partial *R*^2^) of their variations, respectively, followed by refractive error (IOPg 0.60%, IOPcc 1.04%) (Table 5).

**Figure 2:**
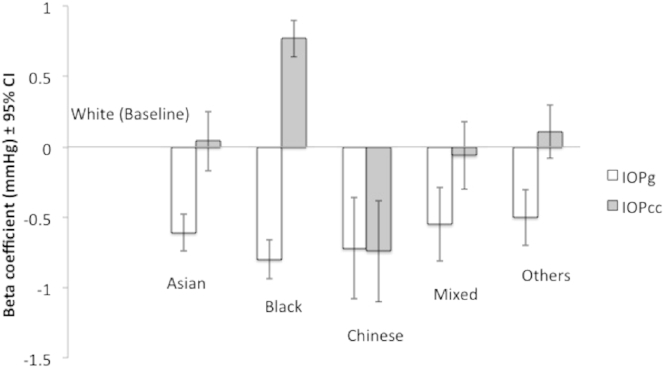
Beta regression coefficients for each ethnic group in the multivariable model, showing the differences between each group's Goldmann-correlated intraocular pressure (IOPg) and corneal-compensated intraocular pressure (IOPcc) compared with the baseline group of white ethnicity. CI = confidence interval.

Some examined factors had different relationships with IOPg and IOPcc in the multivariable model. Self-reported diabetes was significantly associated with IOPg (0.41 mmHg, *P <* 0.001) but not with IOPcc (−0.05 mmHg, *P* = 0.38). The following covariates had different directions of association with IOPg and IOPcc: height (−0.77 mmHg/m IOPg, *P <* 0.001; 1.03 mmHg/m IOPcc, *P <* 0.001), smoking (0.19 mmHg IOPg, *P <* 0.001; −0.35 mmHg IOPcc, *P <* 0.001), and ethnicity, where IOPg was highest among whites (baseline) and lowest among blacks (−0.80 mmHg, *P <* 0.001), but IOPcc was highest among blacks (0.77 mmHg, *P <* 0.001) and lowest among the Chinese (−0.74 mmHg, *P <* 0.001) (Fig 2). This suggests that height, smoking, and ethnicity are strongly related to corneal biomechanical properties. The same set of covariates explained 7.4% of the variability of IOPcc, but only 5.3% of the variability of IOPg.

**Figure 3:**
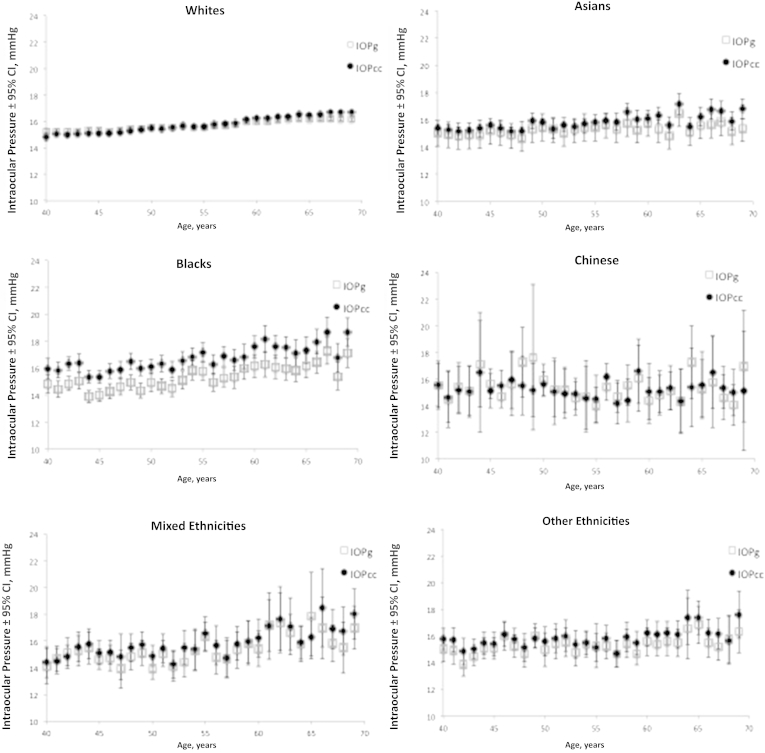
Variation of Goldmann-correlated intraocular pressure (IOPg) and corneal-compensated intraocular pressure (IOPcc) with age for each ethnic group. The error bars represent the 95% confidence intervals (CIs).

The association of IOP and age was examined for each ethnic group. Figure 3 demonstrates how changes in mean IOP with age varies across the ethnic groups, showing a linear increase among whites, Asians, blacks, and those of mixed ethnicities, and the trends are similar between IOPg and IOPcc. The increase was greatest among those of mixed ethnicities after adjusting for covariates (mixed 0.55 mmHg IOPg/decade, 0.64 mmHg IOPcc/decade), followed by black participants (0.42 mmHg IOPg/decade, 0.54 mmHg IOPcc/decade) (Table 6). There was no statistically significant trend among Chinese and “other” ethnicities for IOP and age.

Sensitivity analysis using right eye IOP values and right eye–specific variables (e.g., refraction) was performed for the regression analysis. The only different results were for sex, which was no longer significantly associated with IOPg (0.09 mmHg; 95% CI, 0.03–0.16 mmHg; *P* = 0.007), and with BMI, which was no longer significant with IOPcc (−0.005 mmHg, 95% CI, −0.011 to 0.001 mmHg; *P* = 0.11).

## Discussion

We examined the physical and demographic associations with IOP in one of the largest cohort studies in recent years. This is also one of the few studies that examined and contrasted the associations of IOPg and IOPcc together in a large cohort.

### Goldmann-Correlated Intraocular Pressure versus Corneal-Compensated Intraocular Pressure

In this study, the associations of most variables with IOPg and IOPcc were similar. However, after adjusting for confounders, there were clear differences in the association of IOPg and IOPcc with self-reported diabetes (positively and significantly associated with IOPg but not with IOPcc), height (positively associated with IOPcc, negatively associated with IOPg), smoking (positively associated with IOPg but negatively associated with IOPcc), and black ethnicity (negatively associated with IOPg, positively associated with IOPcc). Previous studies using Goldmann applanation tonometry found higher IOP to be associated with self-reported diabetes,^8^, ^12^ whereas no association had been found with height, including 1 study that used IOPg.^9^, ^12^, ^18^ A recent study comparing ORA data among 2 groups of diabetic patients (HbA1c <7%, HbA1c ≥7%) and healthy controls did demonstrate similar differential associations and found that IOPcc was not significantly different among the 3 groups, whereas IOPg was significantly higher in the diabetic patients than in the controls.^19^ For smoking, findings have been variable, with some studies reporting no association^8^, ^9^, ^11^, ^13^, ^18^ and other studies reporting higher IOP in smokers.^10^, ^20^ Among women, IOPg in this study was not significantly associated with smoking in univariable (*P* = 0.004) or multiple regression (*P* = 0.41, not shown in tables), a finding also seen in the Gutenberg Health Study using noncontact tonometry.^21^

The differential systemic associations of IOPg and IOPcc demonstrated probably mean these 2 IOP measures reflect different biological features. The IOPg is calibrated against the Goldmann applanation tonometer, whereas IOPcc is derived by modeling IOP of patients who underwent laser-assisted in situ keratomileusis to minimize the difference in measured pressure before and after surgery,^17^ therefore reflecting an IOP measure with minimal influence from corneal biomechanics.^16^ In particular, central corneal thickness (CCT) is correlated with IOPg but not IOPcc, and IOPcc is not correlated with corneal resistance factor.^17^ However, it is not clear exactly which parameters of corneal biomechanics best describe the difference between IOPg and IOPcc.

Height is related to a longer axial length, deeper anterior chamber, and flatter cornea.^22^, ^23^ Therefore, height is plausibly related to determinants of collagen-related processes, which may explain the different associations with IOPg and IOPcc. There is a clear trend for men being taller than women in our study, and that resulted in paradoxical results in the univariable analysis when the sexes were separated. This was resolved when sex was adjusted for in the model. Chronic high serum glucose in diabetes and the toxicity from smoking could directly influence the cornea to cause the differential associations with IOPg and IOPcc. Diabetes is known to cause corneal epithelial and endothelial dysfunction and thickening of the basement membrane, postulated to occur from advanced glycation end products and changes in the polyol pathway.^24^ Although the damage of cigarette smoke on the cornea is rarely examined, smoking induces oxidative stress on lens protein and the retina, thought to be related to cataract formation and increased risk of age-related macular degeneration.^25^ These tissue effects could be replicated in the cornea.

Overall, the list of systemic and ocular factors examined explained only a small proportion of IOPg and IOPcc variation (adjusted *R*^2^: 5.3% IOPg, 7.4% IOPcc). Other published studies reported similarly low explanatory power in their models (*R*^2^ of 10.19%–11.0% using Goldmann IOP),^5^, ^6^, ^7^, ^8^ although the list of explanatory variables varies greatly among studies, and therefore the *R*^2^ values cannot be directly compared. Nevertheless, the power of large population studies is to allow small effects to be detected, and these small effects could be biologically important. By focusing on the magnitudes of association, self-reported glaucoma has the greatest effect on IOP (β=1.97 mmHg IOPg, 2.30 mmHg IOPcc), which is equivalent to a 5- to 10-fold effect on IOP compared with a decade increase in age (β=0.18 mmHg IOPg, 0.49 mmHg IOPcc). It is also notable that the effect of seasonal change in IOP between winter and summer (β=−0.27 mmHg IOPg, −0.37 mmHg IOPcc) is comparable to the difference in IOP between women and men (β=0.18 mmHg IOPg, 0.35 IOPcc), as well as the difference between smokers and nonsmokers (β=0.19 mmHg IOPg, −0.35 mmHg IOPcc).

### Ethnicity

For ethnicity, the differential associations with IOPg and IOPcc could be related to ethnic differences in corneal hysteresis or CCT. Thick or thin CCT is known to cause overestimation or underestimation, respectively, of the true IOP by Goldmann applanation tonometers and corneal curvature.^14^ Studies have consistently found CCT to be thinner in Africans than in white subjects,^26^, ^27^ which supports our findings of significantly higher (by 0.80 mmHg) IOPg in whites than blacks, but the opposite in IOPcc (by 0.77 mmHg) once the thinner cornea is taken into account.

Few studies directly compared IOP between ethnic groups. With the large number of participants in the UK Biobank, we were also able to demonstrate that white participants had significantly higher IOPg than Asians, Chinese, and those with mixed or other ancestries. However, with IOPcc, the differences between whites and Asians, and those with mixed and other ancestries, were no longer significant, indicating that corneal biomechanics attenuated the observed differences in IOPg. Of note, Chinese participants had the lowest IOPcc and the second-lowest IOPg, indicating little attenuation by corneal biomechanics. This corroborates with the finding that the IOPg and IOPcc among Chinese participants showed no statistical difference (*P* = 0.52, paired *t* test), whereas all other ethnic groups showed significant differences (all *P <* 0.001, paired *t* test).

### Age

Studies in the past have found inconsistent relationships of IOP with age in regression analyses, ranging from a positive association,^7^, ^8^, ^9^, ^10^, ^18^, ^28^ an inverse relationship,^11^, ^12^ and no association.^6^, ^13^, ^29^ For subjects aged 40 to 69 years, this study found a positive relationship with IOP, which persisted after adjusting for confounders. The Beijing Eye study found IOP increasing up to age 60 to 64 years and decreasing thereafter to age 75 years.^30^ The EPIC-Norfolk Eye Study also found the same trend among women,^18^ and there is a hint of the same “inverted U” trend in our data as IOPg reaches a plateau at age 65 (Fig 1A), although there were no data beyond age 69 years. Corneal-compensated IOP continues to increase at age 65 years or more, and this trend mirrors the increasing prevalence of glaucoma with age. Age is one of the most important risk factors for open-angle glaucoma (OAG), and the results of this study support the possibility that the effect could be mediated partly by higher IOP in older people. In sensitivity analysis using right eye IOP values, the association with age was less significant with IOPg (*P* = 0.007), although the direction of association remains. It was the introduction of SBP into the model that attenuated the effect of age on right eye IOPg.

The large size of this study allows us to further examine the relationship of age with IOP in different ethnic groups. The increase in both IOPg and IOPcc with age was greatest among those of mixed ethnicities, followed by blacks and whites. The trend among Chinese and “other” ethnicities was not clear, and this in part could be due to these 2 groups having relatively smaller numbers, and the size of the change in IOP per decade of age could inherently be small.

The trend of increase in OAG prevalence with age has been examined in 2 recently published meta-analyses, and both studies found Hispanics to have the steepest rate of increase in OAG cases with age.^31^, ^32^ However, both studies also confirmed that the prevalence of OAG was actually highest among blacks, followed by Hispanics and Asians, and lowest among white subjects.

Although we cannot relate the observations of Hispanics to our study because they were not identified as a separate group, it seems that the trend in OAG prevalence found in recent studies mirrors our findings in ethnic differences in IOPcc. Elevated IOPcc could be a useful indicator of OAG risk.

### Sex

We found IOP to be higher in men than in women after adjusting for confounders. This contrasts with several studies that found IOP to be higher in women than in men^7^, ^8^, ^10^, ^18^ or found no difference,^5^, ^9^, ^11^, ^13^, ^30^ but it is supported by 1 study that used noncontact tonometry.^21^ In addition, meta-analyses of the prevalence of OAG have consistently shown men to be 1.36 to 1.37 times more likely than women to have OAG after adjusting for age, race, and study design.^32^, ^33^ A possible reason other studies found different associations with IOP could be their smaller sample sizes, which could be underpowered to detect the difference, although there could be true differences in the populations surveyed.

### Blood Pressure

Systolic blood pressure was the strongest determinant of IOP in this study, which is in agreement with most other studies reporting similar analyses.^7^, ^8^, ^9^, ^10^, ^13^, ^18^, ^30^ Other hemodynamic factors such as DBP^13^, ^30^ and pulse rate^8^, ^10^, ^13^ were also associated with IOP in this study and previous publications. This reflects the dynamic role they have in aqueous production, which is mediated by ciliary blood flow and ciliary oxygen delivery, as well as in regulating aqueous outflow by their effects on episcleral venous pressure and pulse-dependent motion of the trabecular meshwork.^34^

### Body Mass Inde**x**

In the univariable regression model, IOPg and IOPcc had a positive relationship with BMI, but after adjusting for confounders, they were associated with lower BMI (IOPg *P* = 0.009, IOPcc *P <* 0.001). This contrasts with all previous studies that found IOP to be associated with higher BMI,^7^, ^8^, ^10^, ^11^, ^21^, ^35^ even if not statistically significant.^18^ It was the introduction of SBP into the model that switched the direction of association, indicating that SBP was a major confounder in the relationship between IOP and BMI in this study. In the sensitivity analysis using right eye IOP values, a similar attenuation effect was found, where BMI was positively associated with both IOPg and IOPcc in the univariable model (*P <* 0.0001), but the association was no longer significant in the multivariable model (IOPg 0.005 mmHg, 95% CI, −0.0005 to 0.011 mmHg, *P* = 0.073; IOPcc −0.005 mmHg, 95% CI, −0.02 to 0.001 mmHg, *P* = 0.11). Again, it was the introduction of SBP (IOPg) and SBP and pulse rate (IOPcc) into the model that negated the association with BMI.

### Refractive Error

Refractive error was the second most important predictor of both IOPg and IOPcc. Higher IOP was associated with increasing myopic refraction, which persisted even if pseudophakic participants were excluded (results not shown). This corroborates other studies that reported refractive error^8^, ^13^, ^18^, ^30^ and studies that reported IOP increases with longer axial length.^7^, ^12^, ^18^ Myopia is a well-established risk factor for glaucoma,^36^, ^37^, ^38^ although the exact mechanism is unknown. The current results support, at least in part, an IOP-related mechanism.

### Season

The effect of seasonality on IOP has been shown in longitudinal studies in Sweden^39^ and Shanghai,^40^ and among ocular hypertensives in Pakistan^41^ and the United States, as well as cross-sectional population studies in the United States^8^ and Barbados.^10^ These studies all demonstrated higher IOP in the colder months than the warmer months. Our IOPg and IOPcc data also corroborated these findings and showed that the trend is not restricted to applanation tonometry. Temperature, hydration, and daylight hours^41^, ^42^ all have been suggested as possible explanations.

### Center of Assessment

The 6 centers are widely distributed geographically within the United Kingdom, covering the midlands (Birmingham), northern England (Liverpool, Sheffield), Wales (Swansea), and Greater London (Hounslow, Croydon). They each contributed different proportions of subjects to the study cohort, with Swansea accounting for only 0.4% of the study. Participants in these centers also differed in many physical characteristics. Initial analysis of variance analysis showed IOPg to be similar between the centers, but IOPcc was significantly higher in Birmingham than all centers except Swansea. The findings remain in multivariable regression, in which IOPg was similar between the baseline center of Croydon and all other centers, and IOPcc was significantly lower in Birmingham than Croydon. Sensitivity analysis was performed by excluding data from Birmingham, and the main findings of differential associations of IOPg and IOPcc with the physical characteristics remained.

### Study Strengths and Limitations

The strength of this study is the large sample size of 110 573 participants, which is 19 to 55 times larger than to most population studies that reported associations with IOP.^7^, ^9^ This allows weaker associations to be shown. However, the price of achieving such a large sample size efficiently is a low study response rate (5.5%). Together with the volunteer nature and the relatively young age group of <70 years, the study participants are likely to be a healthier sample of the UK population, and therefore are unrepresentative of the general population. Nevertheless, a diverse range of exposures and characteristics are likely to have been captured in such a large study, such that the results reported can still be applicable to other populations with a different distribution of these exposures. The self-reported nature of diabetes, glaucoma, and macular degeneration could affect the observed associations because of recall and misclassification errors. However, more advanced diseases were likely to be included and to bias the outcome by increasing the likelihood of an association being found.

Another limitation of this study is that only 1 IOP measurement was made for each participant, rendering the data more prone to measurement error than if multiple measurements were taken. However, it is reassuring that the standard deviation of 3.8 to 3.9 mmHg for IOP in this study is comparable to 3.7 mmHg reported in another population study using the ORA, which used an average of 3 measurements.^18^

In conclusion, this is the largest study of associations of IOP with demographic and systemic factors to date. It has confirmed many known associations and demonstrated previously unknown differential associations with IOPg and IOPcc. The findings provide insight into the relationship between corneal biomechanics with systemic factors and their effect on IOP measurements.

## Figures and Tables

**Table 1:** Intraocular Pressure Stratified By Age, Sex, and Eye

	IOPg, mmHg (SD, 95% CI)		IOPcc, mmHg (SD, 95% CI)	
Right (n=111 434)	Left (n=111 049)	Right (n=111 434)	Left (n=111 049)
Men				
40–49 yrs	15.4 (3.7, 15.4–15.5)	15.4 (3.9, 15.3–15.4)	15.6 (3.6, 15.5–15.6)	15.6 (3.7, 15.5–15.6)
50–59 yrs	15.9 (4.0, 15.8–16.0)	15.7 (3.9, 15.7–15.8)	16.1 (3.9, 16.1–16.2)	16.1 (3.9, 16.0–16.1)
60–69 yrs	16.3 (4.0, 16.2–16.3)	16.2 (4.1, 16.1–16.2)	16.8 (4.0, 16.7–16.8)	16.7 (4.1, 16.7–16.8)
Women				
40–49 yrs	15.3 (3.6, 15.2–15.3)	15.0 (3.5, 14.9–15.0)	15.0 (3.5, 15.0–15.1)	14.9 (3.4, 14.8–15.0)
50–59 yrs	15.6 (3.7, 15.6–15.7)	15.4 (3.8, 15.4–15.5)	15.6 (3.6, 15.5–15.6)	15.4 (3.7, 15.4–15.5)
60–69 yrs	16.2 (3.8, 16.1–16.2)	16.0 (3.9, 16.0–16.1)	16.3 (3.8, 16.3–16.4)	16.2 (3.9, 16.2–16.3)
Total				
40–49 yrs	15.3 (3.7, 15.3–15.4)	15.2 (3.7, 15.1–15.2)	15.3 (3.5, 15.2–15.3)	15.2 (3.6, 15.1–15.2)
50–59 yrs	15.8 (3.9, 15.7–15.8)	15.6 (3.8, 15.5–15.6)	15.8 (3.8, 15.77–15.85)	15.7 (3.8, 15.7–15.8)
60–69 yrs	16.2 (3.9, 16.2–16.2)	16.1 (4.0, 16.1–16.1)	16.5 (3.9, 16.5–16.6)	16.5 (4.0, 16.45–16.52)
All	15.86 (3.8, 15.84–15.88)	15.72 (3.9, 15.70–15.74)	16.02 (3.8, 16.00–16.04)	15.95 (3.9, 15.92–15.97)
Difference (right-left)	0.14 (0.12–0.16), *P* < 0.001		0.07 (0.05–0.09), *P* < 0.001	

CI = confidence interval; IOPcc = corneal-compensated intraocular pressure; IOPg = Goldmann-correlated intraocular pressure; SD = standard deviation.

Data shown are for 112 690 participants in the whole UK Biobank cohort with valid IOP measurements. The IOP values shown are the mean for each age group. The *t* test compares the difference between left and right eye values.

**Table 2:** Characteristics of the 110 573 Study Participants in the UK Biobank, by Sex

	Total	Women	Men	*P* Value∗
Sex, % women		54.1		
Age, yrs (n=110 573)	57.3 (8.1)	57.1 (8.0)	57.6 (8.2)	**<0.001**
Ethnicity, % (n=110 573)				
White	89.6	89.4	89.9	**<0.001**
Asian	3.9	3.4	4.5	
Black	3.6	3.9	3.2	
Chinese	0.46	0.55	0.36	
Mixed	0.90	1.0	0.74	
Others	1.6	1.7	1.3	
Assessment center, % (n= 110 573)				
Croydon	22.9	23.8	21.8	**<0.001**
Sheffield	22.4	22.0	22.8	
Birmingham	21.6	21.0	22.2	
Hounslow	18.6	18.9	18.3	
Liverpool	14.2	14.0	14.4	
Swansea	0.39	0.36	0.43	
Townsend deprivation index (n=110 438)	−0.94 (3.0)	−0.96 (3.0)	−0.91 (3.1)	0.004
Height, m (n=110 127)	1.69 (0.09)	1.63 (0.06)	1.76 (0.07)	**<0.001**
Weight, kg (n=110 102)	78.2 (16.1)	71.6 (14.2)	86.0 (14.5)	**<0.001**
BMI, kg/m^2^ (n=105 113)	27.4 (4.8)	27.1 (5.2)	27.8 (4.3)	**<0.001**
Waist circumference, cm (n=110 268)	90.6 (13.6)	85.1 (12.7)	97.1 (11.5)	**<0.001**
SBP, mmHg (n=110 510)	137.4 (18.4)	135.1 (19.0)	140.0 (17.2)	**<0.001**
DBP, mmHg (n=110 510)	81.9 (10.0)	80.5 (9.9)	83.6 (9.9)	**<0.001**
Pulse rate, min^−1^ (n=110 510)	68.6 (11.1)	69.4 (10.4)	67.7 (11.8)	**<0.001**
Refractive error, D				
Right eye (n=1 109 376)	−0.36 (2.8)	−0.31 (2.8)	−0.35 (2.7)	0.14
Left eye (n=109 059)	−0.31 (2.8)	−0.31 (2.8)	−0.30 (2.7)	0.42
Current smoking status, % (n=107 115)				
Regular smoker	7.2	6.2	8.5	**<0.001**
Occasional smoker	2.8	2.1	3.7	
Nonsmoker	90.0	91.7	87.9	
Self-reported diabetes, % (n=109 832)	5.9	4.4	7.7	**<0.001**
Self-reported glaucoma, %				
Right eye (n=110 573)	1.46	1.17	1.80	**<0.001**
Left eye (n=110 573)	1.45	1.14	1.82	**<0.001**
Self-reported macular degeneration, %				
Right eye (n=110 573)	0.85	0.92	0.77	0.006
Left eye (n=110 573)	0.82	0.90	0.72	0.001
Season of test, % (n=110 573)				
Spring	35.0	34.9	35.25	0.040
Summer	19.9	20.1	19.6	
Autumn	23.1	23.2	22.95	
Winter	22.0	21.8	22.2	

BMI = body mass index; D = diopters; DBP = diastolic blood pressure; SBP = systolic blood pressure.

The study participants excluded those who had undergone laser refractive surgery or corneal graft surgery in their left eye. For continuous variables, the values shown are mean (standard deviation).

∗ *P* values are from the *t* test or chi-square test comparing characteristics between men and women in the study cohort. *P* < 0.001 shown in bold.

**Table 3:** Univariable Linear Regression with Goldmann-Correlated Intraocular Pressure (Left Eye) as the Dependent Variable

	All		Women		Men	
β (95% CI)	P	β (95% CI)	P	β (95% CI)	P
Age, decade	0.45 (0.43–0.48)	**<0.001**	0.50 (0.46–0.53)	**<0.001**	0.4 (0.36–0.44)	**<0.001**
Sex (baseline = female)	0.25 (0.20–0.30)	**<0.001**	–	–	–	–
Ethnicity (baseline = white)						
Asian	−0.52 (−0.64 to −0.40)	**<0.001**	−0.65 (−0.82 to −0.48)	**<0.001**	−0.44 (−0.61 to −0.27)	**0.001**
Black	−0.71 (−0.83 to −0.58)	**<0.001**	−0.83 (−0.99 to −0.67)	**<0.001**	−0.50 (−0.70 to −0.30)	**0.001**
Chinese	−0.55 (−0.90 to −0.21)	0.001	−0.72 (−1.14 to −0.31)	0.001	−0.19 (−0.78 to 0.40)	0.53
Mixed	−0.58 (−0.82 to −0.34)	**<0.001**	−0.58 (−0.88 to −0.28)	**<0.001**	−0.52 (−0.93 to −0.11)	0.012
Others	−0.58 (−0.77 to −0.39)	**<0.001**	−0.69 (−0.93 to −0.46)	**<0.001**	−0.37 (−0.68 to −0.068)	0.017
Assessment center (baseline = Croydon)						−
Sheffield	0.088 (0.20–0.16)	0.012	0.13 (0.045–0.22)	0.003	0.018 (−0.087 to 0.12)	0.73
Birmingham	0.011 (−0.06 to 0.08)	0.76	0.008 (−0.08 to 0.10)	0.87	−0.052 (−0.11 to 0.09)	0.79
Hounslow	0.044 (−0.028 to 0.12)	0.23	0.015 (−0.08 to 0.11)	0.76	0.072 (−0.040 to 0.18)	0.21
Liverpool	0.14 (0.057–0.21)	0.001	0.081 (−0.022 to 0.18)	0.12	0.18 (0.062–0.30)	0.003
Swansea	0.19 (−0.18 to 0.57)	0.31	0.20 (−0.31 to 0.71)	0.44	0.15 (−0.39 to 0.70)	0.58
Deprivation index	−0.010 (−0.018 to −0.002)	0.011	−0.024 (−0.034 to −0.013)	**<0.001**	0.005 (−0.007 to 0.016)	0.44
Weight, 10 kg	0.08 (0.062–0.091)	**<0.001**	0.07 (0.04–0.9)	**<0.001**	0.04 (0.01–0.06)	0.003
Height, m	0.09 (−0.16 to 0.34)	0.47	−1.84 (−2.32 to −1.36)	**0.001**	−0.15 (−0.20 to −0.10)	**<0.001**
BMI, kg/m^2^	0.033 (0.027–0.038)	**<0.001**	0.031 (0.024–0.039)	**<0.001**	0.028 (0.019–0.038)	**<0.001**
Waist, cm	0.014 (0.013–0.016)	**<0.001**	0.014 (0.012–0.016)	**<0.001**	0.011 (0.008–0.015)	**<0.001**
SBP, mmHg	0.039 (0.038–0.040)	**<0.001**	0.037 (0.035–0.038)	**<0.001**	0.041 (0.039–0.043)	**<0.001**
DBP, mmHg	0.054 (0.052–0.056)	**<0.001**	0.053 (0.050–0.056)	**<0.001**	0.055 (0.051–0.058)	**<0.001**
Pulse, min^−1^	0.030 (0.028–0.032)	**<0.001**	0.036 (0.033–0.039)	**<0.001**	0.027 (0.024–0.030)	**<0.001**
Refractive error, D	−0.085 (−0.093 to −0.077)	**<0.001**	−0.073 (−0.084 to −0.062)	**<0.001**	−0.10 (−0.11 to −0.088)	**<0.001**
Smoking						
Nonsmoker = 0	–	–	–	–	–	–
Regular smoker = 1	0.12 (0.026–0.20)	0.011	−0.049 (−0.18 to 0.08)	0.45	0.22 (0.09–0.34)	0.001
Diabetes	0.69 (0.60–0.79)	**<0.001**	0.86 (0.71–1.00)	**<0.001**	0.52 (0.39–0.65)	**<0.001**
Glaucoma	2.34 (2.15–2.53)	**<0.001**	2.54 (2.25–2.83)	**<0.001**	2.15 (1.88–2.41)	**<0.001**
Macular degeneration	0.51 (0.26–0.80)	**<0.001**	0.34 (0.013–0.66)	0.041	0.81 (0.39–1.22)	**0.001**
Seasons (baseline = winter)						
Spring	−0.20 (−0.26 to −0.14)	**<0.001**	−0.10 (−0.19 to −0.021)	0.014	−0.31 (−0.40 to −0.21)	**<0.001**
Summer	−0.43 (−0.50 to −0.36)	**<0.001**	−0.27 (−0.36 to −0.17)	**<0.001**	−0.62 (−0.73 to −0.51)	**<0.001**
Autumn	−0.13 (−0.19 to −0.57)	**<0.001**	−0.049 (−0.14 to 0.042)	0.29	−0.21 (−0.31 to −0.10)	**<0.001**

BMI = body mass index; CI = confidence interval; D = diopters; DBP = diastolic blood pressure; IOPg = Goldmann-correlated intraocular pressure; SBP = systolic blood pressure.

*P <* 0.001 shown in bold. BMI between 20 and 40 kg/m^2^ was analyzed.

**Table 4:** Univariable Linear Regression with Corneal-Compensated Intraocular Pressure (Left Eye) as the Dependent Variable

	All		Women		Men	
β (95% CI)	P	β (95% CI)	P	β (95% CI)	P
Age, decade	0.67 (0.64–0.70)	**<0.001**	0.70 (0.67–0.73)	**<0.001**	0.61 (0.57–0.65)	**<0.001**
Sex (female = 0, male = 1)	0.61 (0.56–0.66)	**<0.001**	–	–	–	–
Ethnicity (baseline = white)						
Asian	−0.16 (−0.28 to −0.04)	0.009	−0.25 (−0.42 to −0.08)	0.004	−0.15 (−0.32 to 0.02)	0.08
Black	0.56 (0.44–0.69)	**<0.001**	0.49 (0.33–0.65)	**<0.001**	0.74 (0.54–0.94)	**<0.001**
Chinese	−0.77 (−1.11 to −0.44)	**<0.001**	−0.80 (−1.21 to −0.39)	**<0.001**	−0.55 (−1.13 to 0.03)	0.06
Mixed	−0.31 (−0.55 to −0.07)	0.013	−0.18 (−0.48 to 0.12)	0.23	−0.38 (−0.79 to 0.02)	0.06
Others	−0.22 (−0.41 to −0.035)	0.020	−0.13 (−0.36 to 1.02)	0.27	−0.26 (−0.57 to 0.04)	0.09
Center of assessment (baseline = Croydon)						
Sheffield	0.053 (−0.015 to 0.12)	0.13	0.028 (−0.06 to 0.12)	0.53	0.042 (−0.061 to 0.15)	0.42
Birmingham	−0.37 (−0.44 to −0.30)	**<0.001**	−0.39 (−0.48 to −0.30)	**<0.001**	−0.40 (−0.51 to −0.30)	**<0.001**
Hounslow	−0.014 (−0.086 to 0.057)	0.69	−0.042 (−0.13 to 0.051)	0.38	0.002 (−0.11 to 0.11)	0.98
Liverpool	0.042 (−0.036 to 0.12)	0.29	−0.041 (−0.14 to 0.061)	0.43	0.098 (−0.02 to 0.22)	0.10
Swansea	0.065 (−0.30 to 0.43)	0.73	0.19 (−0.32 to 0.70)	0.46	−0.13 (−0.67 to 0.40)	0.63
Deprivation index	−0.02 (−0.03 to −0.01)	**<0.001**	−0.02 (−0.03 to −0.01)	**<0.001**	−0.02 (−0.04 to −0.01)	**<0.001**
Weight, 10 kg	0.12 (0.10–0.13)	**<0.001**	0.08 (0.06–0.10)	**<0.001**	−0.0004 (−0.02 to 0.02)	0.10
Height, m	2.02 (1.77–2.23)	**<0.001**	−0.91 (−1.38 to −0.43)	**<0.001**	−0.18 (−0.69 to 0.33)	0.48
BMI, kg/m^2^	0.025 (0.019–0.030)	**<0.001**	0.030 (0.023–0.037)	**<0.001**	−0.0008 (−0.01 to 0.009)	0.87
Waist, cm	0.018 (0.017–0.20)	**<0.001**	0.015 (0.013–0.017)	**<0.001**	0.004 (0.001–0.007)	0.007
SBP, mmHg	0.042 (0.040–0.043)	**<0.001**	0.040 (0.039–0.042)	**<0.001**	0.040 (0.038–0.042)	**<0.001**
DBP, mmHg	0.058 (0.056–0.061)	**<0.001**	0.057 (0.054–0.060)	**<0.001**	0.053 (0.049–0.056)	**<0.001**
Pulse, min^−1^	0.021 (0.019–0.023)	**<0.001**	0.031 (0.028–0.033)	**<0.001**	0.016 (0.013–0.019)	**<0.001**
Refractive error, D	−0.11 (−0.12 to −0.11)	**<0.001**	−0.63 (−0.75 to −0.50)	**<0.001**	−0.14 (−0.15 to −0.13)	**<0.001**
Smoking						
Nonsmoker = 0	−	−	−	−	−	
Regular smoker = 1	−0.51 (−0.60 to −0.42)	**<0.001**	−0.61 (−0.74 to −0.49)	**<0.001**	−0.51 (−0.64 to −0.39)	**<0.001**
Diabetes	0.38 (0.28–0.48)	**<0.001**	0.53 (0.38–0.68)	**<0.001**	0.13 (−0.03 to 0.26)	0.06
Glaucoma	2.34 (2.15–2.53)	**<0.001**	2.84 (2.55–3.13)	**<0.001**	2.74 (2.48–3.00)	**<0.001**
Macular degeneration	0.79 (0.53–1.04)	**<0.001**	0.63 (0.31–0.95)	**<0.001**	1.11 (0.69–1.51)	**<0.001**
Seasons (baseline = winter)						
Spring	−0.36 (−0.42 to −0.29)	**<0.001**	−0.23 (−0.31 to −0.14)	**<0.001**	−0.50 (−0.60 to −0.41)	**<0.001**
Summer	−0.56 (−0.62 to −0.48)	**<0.001**	−0.40 (−0.49 to −0.30)	**<0.001**	−0.73 (−0.83 to −0.62)	**<0.001**
Autumn	0.011 (−0.06 to 0.08)	0.75	0.12 (0.028–0.21)	0.011	−0.10 (−0.21 to 0.0008)	0.05

BMI = body mass index; CI = confidence interval; D = dipoters; DBP = diastolic blood pressure; SBP = systolic blood pressure.

*P <* 0.001 shown in bold. BMI between 20 and 40 kg/m^2^ was analyzed.

**Table 5:** Multivariable Linear Regression with Goldmann-Correlated Intraocular Pressure and Corneal-Compensated Intraocular Pressure (Left Eye) as the Dependent Variables

	IOPg				IOPcc			
β (95% CI)	P	Standard Coefficient∗	Partial R^2^ (%)	β (95% CI)	P	Standard Coefficient∗	Partial R^2^ (%)
Age, decade	0.18 (0.15–0.21)	**<0.001**	0.15	0.12	0.49 (0.46–0.52)	**<0.001**	0.39	0.9
Sex (baseline = female)	0.18 (0.11–0.25)	**<0.001**	n/a	0.03	0.35 (0.28–0.42)	**<0.001**	n/a	0.1
Ethnicity (baseline = white)			n/a				n/a	
Asian	−0.61 (−0.74 to −0.48)	**<0.001**	0.09	0.042 (−0.09 to 0.17)	0.52	0
Black	−0.80 (−0.94 to −0.66)	**<0.001**	0.13	0.77 (0.63–0.90)	**<0.001**	0.13
Chinese	−0.72 (−1.08 to −0.36)	**<0.001**	0.02	−0.74 (−1.10 to −0.38)	**<0.001**		0.02
Mixed	−0.55 (−0.80 to −0.29)	**<0.001**	0.02	−0.06 (−0.30 to 0.19)	0.66	0
Others	−0.50 (−0.70 to −0.30)	**<0.001**	0.02	0.11 (−0.088 to 0.30)	0.28	0
Center of assessment (baseline = Croydon)								
Sheffield	−0.07 (−0.14 to 0.002)	0.058		0	0.012 (−0.06 to 0.83)	0.74		0
Birmingham	−0.056 (−0.13 to 0.017)	0.13	n/a	0	−0.32 (−0.39 to −0.25)	**<0.001**	n/a	0.08
Hounslow	−0.005 (−0.07 to 0.08)	0.89		0	−0.04 (−0.11 to 0.04)	0.32		0
Liverpool	−0.12 (−0.21 to −0.04)	0.005		0.01	−0.15 (−0.23 to −0.06)	0.001		0.01
Swansea	−0.24 (−0.63 to 0.15)	0.23		0	−0.014 (−0.39 0.37)	0.94		0
Deprivation index	0.007 (−0.001 to 0.016)	0.10	0.02	0	−0.004 (−0.013 to 0.004)	0.32	−0.001	0
Height, m	−0.77 (−1.14 to −0.39)	**<0.001**	−0.07	0.02	1.03 (0.65–1.40)	**<0.001**	0.095	0.03
BMI, kg/m^2^	−0.008 (−0.014 to −0.002)	0.009	−0.03	0.01	−0.016 (−0.022 to −0.011)	**<0.001**	−0.067	0.03
SBP, mmHg	0.035 (0.033–0.036)	**<0.001**	0.63	2.29	0.033 (0.032–0.034)	**<0.001**	0.60	2.16
Pulse, min^−1^	0.023 (0.021–0.025)	**<0.001**	0.26	0.43	0.018 (0.016–0.02)	**<0.001**	0.20	0.28
Refractive error, D	−0.11 (−0.12 to −0.10)	**<0.001**	−0.30	0.58	−0.14 (−0.15 to −0.13)	**<0.001**	−0.39	1.04
Smoking (baseline = nonsmoker)								
Regular smoker	0.19 (0.097–0.28)	**<0.001**	n/a	0.02	−0.35 (−0.44 to −0.26)	**<0.001**	n/a	0.06
Self-reported diabetes	0.41 (0.3–0.52)	**<0.001**	n/a	0.06	−0.05 (−0.15 to 0.06)	0.38	n/a	0
Self-reported glaucoma	1.97 (1.77–2.17)	**<0.001**	n/a	0.38	2.30 (2.11–2.50)	**<0.001**	n/a	0.54
Self-reported macular degeneration	0.21 (−0.053 to 0.47)	0.12	n/a	0	0.34 (0.087–0.60)	0.009	n/a	0.01
Seasons (baseline = winter)								
Spring	−0.14 (−0.21 to −0.075)	**<0.001**		0.02	−0.29 (−0.35 to −0.22)	**<0.001**		0.08
Summer	−0.27 (−0.35 to −0.20)	**<0.001**	n/a	0.05	−0.37 (−0.44 to −0.30)	**<0.001**	n/a	0.1
Autumn	−0.04 (−0.11 to 0.03)	0.30		0	0.066 (−0.003 to 0.14)	0.06		0
Adjusted *R*^2^ (%)	5.3				7.4			

BMI = body mass index; CI = confidence interval; D = diopters; DBP = diastolic blood pressure; IOPcc = corneal-compensated intraocular pressure; IOPg = Goldmann-correlated intraocular pressure; n/a = not available; SBP = systolic blood pressure.

∗ For continuous covariates, standardized coefficient represents change in IOP (mmHg) per standard deviation of the covariate. *P <* 0.001 shown in bold. BMI between 20 and 40 kg/m^2^ was analyzed.

**Table 6:** Relationship of Age with Goldmann-Correlated Intraocular Pressure and Corneal-Compensated Intraocular Pressure in Different Ethnic Groups

	IOPg		IOPcc	
β (95% CI)	P Value	β (95% CI)	P Value
White	0.18 (0.15–0.21)	**<0.001**	0.51 (0.47–0.54)	**<0.001**
Asian	0.07 (−0.09 to 0.24)	0.39	0.25 (0.09–0.41)	**<0.001**
Black	0.42 (0.23–0.60)	**<0.001**	0.54 (0.35–0.73)	**<0.001**
Chinese	−0.52 (−1.04 to 0.01)	0.06	−0.37 (−0.84 to 0.11)	0.13
Mixed	0.55 (0.21–0.90)	**<0.001**	0.64 (0.30–0.98)	**<0.001**
Others	0.13 (−0.16 to 0.41)	0.38	0.29 (0.02–0.56)	0.03

CI = confidence interval; IOPcc = corneal-compensated intraocular pressure; IOPg = Goldmann-correlated intraocular pressure.

Shown are regression coefficients for age (mmHg IOP/10 years of age) in multivariable linear models for each ethnic group, with IOPg and IOPcc as dependent variables. The following covariates were used in every model: sex, center of assessment, deprivation index, height, body mass index, systolic blood pressure, pulse, refractive error, smoking status, self-reported diabetes, glaucoma and macular degeneration, and seasons. *P <* 0.001 shown in bold.

## Supplementary Data

UK Biobank Eye and Vision Consortium Member List

## References

[1] de Voogd S., Ikram M.K., Wolfs R.C. (2005). Incidence of open-angle glaucoma in a general elderly population: the Rotterdam Study. Ophthalmology.

[2] Leske M.C., Heijl A., Hyman L. (2007). Predictors of long-term progression in the early manifest glaucoma trial. Ophthalmology.

[3] Carbonaro F., Andrew T., Mackey D.A. (2008). Heritability of intraocular pressure: a classical twin study. Br J Ophthalmol.

[4] Chang T.C., Congdon N.G., Wojciechowski R. (2005). Determinants and heritability of intraocular pressure and cup-to-disc ratio in a defined older population. Ophthalmology.

[5] Weih L.M., Mukesh B.N., McCarty C.A. (2001). Association of demographic, familial, medical, and ocular factors with intraocular pressure. Arch Ophthalmol.

[6] Wang D., Huang W., Li Y. (2011). Intraocular pressure, central corneal thickness, and glaucoma in Chinese adults: the Liwan eye study. Am J Ophthalmol.

[7] Memarzadeh F., Ying-Lai M., Azen S.P. (2008). Associations with intraocular pressure in Latinos: the Los Angeles Latino Eye Study. Am J Ophthalmol.

[8] Klein B.E., Klein R., Linton K.L. (1992). Intraocular pressure in an American community. The Beaver Dam Eye Study. Invest Ophthalmol Vis Sci.

[9] Foster P.J., Machin D., Wong T.-Y. (2003). Determinants of intraocular pressure and its association with glaucomatous optic neuropathy in Chinese Singaporeans: the Tanjong Pagar Study. Invest Ophthalmol Vis Sci.

[10] Wu S.Y., Leske M.C. (1997). Associations with intraocular pressure in the Barbados Eye Study. Arch Ophthalmol.

[11] Kawase K., Tomidokoro A., Araie M. (2008). Ocular and systemic factors related to intraocular pressure in Japanese adults: the Tajimi study. Br J Ophthalmol.

[12] Tomoyose E., Higa A., Sakai H. (2010). Intraocular pressure and related systemic and ocular biometric factors in a population-based study in Japan: the Kumejima study. Am J Ophthalmol.

[13] Jonas J.B., Nangia V., Matin A. (2011). Intraocular pressure and associated factors: the central India eye and medical study. J Glaucoma.

[14] Doughty M.J., Zaman M.L. (2000). Human corneal thickness and its impact on intraocular pressure measures: a review and meta-analysis approach. Surv Ophthalmol.

[15] Liu J., Roberts C.J. (2005). Influence of corneal biomechanical properties on intraocular pressure measurement: quantitative analysis. J Cataract Refract Surg.

[16] Medeiros F.A., Weinreb R.N. (2006). Evaluation of the influence of corneal biomechanical properties on intraocular pressure measurements using the ocular response analyzer. J Glaucoma.

[17] Luce D. (2006). Methodology for cornea compensated IOP and corneal resistance factor for the Reichert Ocular Response Analyzer. Invest Ophthalmol Vis Sci.

[18] Foster P.J., Broadway D.C., Garway-Heath D.F. (2011). Intraocular pressure and corneal biomechanics in an adult British population: the EPIC-Norfolk eye study. Invest Ophthalmol Vis Sci.

[19] Yazgan S., Celik U., Kaldirim H. (2014). Evaluation of the relationship between corneal biomechanic and HbA1C levels in type 2 diabetes patients. Clin Ophthalmol.

[20] Lee A.J., Rochtchina E., Wang J.J. (2003). Does smoking affect intraocular pressure? Findings from the Blue Mountains Eye Study. J Glaucoma.

[21] Hoehn R., Mirshahi A., Hoffmann E.M. (2013). Distribution of intraocular pressure and its association with ocular features and cardiovascular risk factors: the Gutenberg Health Study. Ophthalmology.

[22] Nangia V., Jonas J.B., Matin A. (2010). Body height and ocular dimensions in the adult population in rural Central India. The Central India Eye and Medical Study. Graefes Arch Clin Exp Ophthalmol.

[23] Wong T.Y., Foster P.J., Johnson G.J. (2001). The relationship between ocular dimensions and refraction with adult stature: the Tanjong Pagar Survey. Invest Ophthalmol Vis Sci.

[24] Kaji Y. (2005). Prevention of diabetic keratopathy. Br J Ophthalmol.

[25] Galor A., Lee D.J. (2011). Effects of smoking on ocular health. Curr Opin Ophthalmol.

[26] Brandt J.D., Beiser J.A., Kass M.A. (2001). Central corneal thickness in the Ocular Hypertension Treatment Study (OHTS). Ophthalmology.

[27] Detry-Morel M., Jamart J., Hautenauven F. (2012). Comparison of the corneal biomechanical properties with the Ocular Response Analyzer (ORA) in African and Caucasian normal subjects and patients with glaucoma. Acta Ophthalmol.

[28] Rochtchina E., Mitchell P., Wang J.J. (2002). Relationship between age and intraocular pressure: the Blue Mountains Eye Study. Clin Experiment Ophthalmol.

[29] Wong T.T., Wong T.Y., Foster P.J. (2009). The relationship of intraocular pressure with age, systolic blood pressure, and central corneal thickness in an Asian population. Invest Ophthalmol Vis Sci.

[30] Xu L., Li J., Zheng Y. (2005). Intraocular pressure in Northern China in an urban and rural population: the Beijing eye study. Am J Ophthalmol.

[31] Kapetanakis V.V., Chan M.P.Y., Foster P.J. (2016). Global variations and time trends in the prevalence of primary open angle glaucoma (POAG): a systematic review and meta-analysis. Br J Ophthalmol.

[32] Tham Y.C., Li X., Wong T.Y. (2014). Global prevalence of glaucoma and projections of glaucoma burden through 2040: a systematic review and meta-analysis. Ophthalmology.

[33] Rudnicka A.R., Mt-Isa S., Owen C.G. (2006). Variations in primary open-angle glaucoma prevalence by age, gender, and race: a Bayesian meta-analysis. Invest Ophthalmol Vis Sci.

[34] Ramos R.F., Sumida G.M., Stamer W.D. (2009). Cyclic mechanical stress and trabecular meshwork cell contractility. Invest Ophthalmol Vis Sci.

[35] Mori K., Ando F., Nomura H. (2000). Relationship between intraocular pressure and obesity in Japan. Int J Epidemiol.

[36] Xu L., Wang Y., Wang S. (2007). High myopia and glaucoma susceptibility the Beijing Eye Study. Ophthalmology.

[37] Wong T.Y., Klein B.E., Klein R. (2003). Refractive errors, intraocular pressure, and glaucoma in a white population. Ophthalmology.

[38] Grodum K., Heijl A., Bengtsson B. (2001). Refractive error and glaucoma. Acta Ophthalmol Scand.

[39] Bengtsson B. (1972). Some factors affecting the distribution of intraocular pressures in a population. Acta Ophthalmol (Copenh).

[40] Qureshi I.A., Xi X.R., Lu H.J. (1996). Effect of seasons upon intraocular pressure in healthy population of China. Korean J Ophthalmol.

[41] Qureshi I.A., Xiao R.X., Yang B.H. (1999). Seasonal and diurnal variations of ocular pressure in ocular hypertensive subjects in Pakistan. Singapore Med J.

[42] Stoupel E., Goldenfeld M., Shimshoni M. (1993). Intraocular pressure (IOP) in relation to four levels of daily geomagnetic and extreme yearly solar activity. Int J Biometeorol.

