# Supplementary material for: Associations with Intraocular Pressure in a Large Cohort: Results from the UK Biobank
Source: Ophthalmology. 2016 Apr;123(4):771–82. doi: 10.1016/j.ophtha.2015.11.031 (PMC4819446; doi:10.1016/j.ophtha.2015.11.031)
Supplement: UK Biobank Eye and Vision Consortium Member List [file mmc1.pdf]

UK Biobank Eye and Vision Consortium member list (correct on 16th March 2015)

Steering Committee

Chair: Prof Andrew Lotery

Co-chair: Prof Andrew Dick

Prof Paul Bishop

Prof Bal Dhillon

Prof Simon Harding

Prof Sir Peng Tee Khaw

Prof James Morgan

Members

Mr Tariq ASLAM - Manchester

Ms Yanchun BAO - University of Essex

Dr Sarah Barman - Kingston University

Prof Paul BISHOP - Manchester University

Mr Peter BLOWS, Moorfields Eye Hospital, London

Prof Usha CHAKRAVARTHY - Queens University, Belfast

Miss Michelle CHAN - Moorfields Eye Hospital, London

Mrs Antonietta CHIANCA - UCL Institute of Ophthalmology

Dr Valentina CIPRIANI - UCL Institute of Ophthalmology

Prof David CRABB - City University, London

Mrs Philippa CUMBERLAND - UCL Institute of Child Health

Dr Alexander DAY – Moorfields Eye Hospital, London

Miss Parul DESAI - Moorfields Eye Hospital, London

Prof Bal DHILLON - Edinburgh University

Prof Andrew DICK - University of Bristol

Prof Paul FOSTER - UCL Institute of Ophthalmology

Dr John GALLACHER - Cardiff University

Prof David (Ted) GARWAY-HEATH - UCL Institute of Ophthalmology

Mrs Priyal GUPTA - UCL Institute of Ophthalmology

Prof Chris HAMMOND - King's College London

Dr Ruth HOGG - Queen's University, Belfast

Prof Anne HUGHES - Queen's University, Belfast

Mr Pearse KEANE - UCL Institute of Ophthalmology

Prof Sir Peng Tee KHAW - UCL Institute of Ophthalmology

Mr Anthony KHAWAJA - Moorfields Eye Hospital, London

Mr Gerassimos LASCARATOS - Moorfields Eye Hospital, London

Prof Andrew LOTERY- University of Southampton
Prof Phil LUTHERT - UCL Institute of Ophthalmology
Dr Tom MACGILLIVRAY - University of Edinburgh
Dr Sarah MACKIE - St James's University Hospital, Leeds
Prof Keith MARTIN - University of Cambridge
Ms Michelle MCGAUGHEY - Queen's University Belfast
Dr Bernadette MCGUINNESS - Queen's University Belfast
Dr Gareth MCKAY - Queen's University Belfast
Mr Martin MCKIBBIN - Leeds Teaching Hospitals NHS Trust
Dr Danny MITRY - University of Edinburgh
Prof Tony MOORE - UCL Institute of Ophthalmology
Prof James MORGAN - Cardiff University
Miss Zaynah MUTHY - UCL Institute of Ophthalmology
Mr Eoin O'SULLIVAN - University of Cambridge
Dr Chris OWEN - St George's, University of London
Mr Praveen PATEL - Moorfields Eye Hospital, London
Dr Tunde PETO - Moorfields Eye Hospital, London
Prof Jugnoo RAHI - UCL Institute of Child Health
Dr Alicja RUDNICKA - St George's, University of London
Miss Carlota Grossi SAMPEDRO - University of East Anglia
Mr David STEEL - Newcastle University
Mrs Irene STRATTON - Gloucestershire Hospitals NHS Foundation Trust
Mr Nicholas STROUTHDIS - Moorfields Eye Hospital, London
Prof Cathie SUDLOW - University of Edinburgh
Miss Dhanes THOMAS - Moorfields Eye Hospital, London
Prof Emanuele TRUCCO - University of Dundee
Mr Adnan TUFAIL - Moorfields Eye Hospital, London
Prof Stephen VERNON - University Hospital, Nottingham
Mr Ananth VISWANATHAN - Moorfields Eye Hospital, London
Miss Cathy WILLIAMS - University of Bristol
Dr Katie WILLIAMS - King's College London
Prof John YATES - University of Cambridge
Dr Max YATES - University of East Anglia
Ms Jennifer YIP - University of Cambridge
Dr Haogang ZHU - City University, London
